# *Phytophthora infestans*: An Overview of Methods and Attempts to Combat Late Blight

**DOI:** 10.3390/jof7121071

**Published:** 2021-12-13

**Authors:** Artemii A. Ivanov, Egor O. Ukladov, Tatiana S. Golubeva

**Affiliations:** 1 Institute of Cytology and Genetics SB RAS, 630090 Novosibirsk, Russia; a.ivanov2@g.nsu.ru; 2 Faculty of Natural Sciences, Novosibirsk State University, 630090 Novosibirsk, Russia; egor_ukladov23579@mail.ru

**Keywords:** late blight, *Phytophthora infestans*, R-genes, pathogen resistance, potato, *Solanum tuberosum*

## Abstract

*Phytophthora infestans* (Mont.) de Bary is one of the main pathogens in the agricultural sector. The most affected are the *Solanaceae* species, with the potato (*Solanum tuberosum*) and the tomato (*Solanum lycopersicum*) being of great agricultural importance. Ornamental *Solanaceae* can also host the pests *Petunia* spp., *Calibrachoa* spp., as well as the wild species *Solanum dulcamara*, *Solanum sarrachoides*, etc. Annual crop losses caused by this pathogen are highly significant. Although the interaction between *P. infestans* and the potato has been investigated for a long time, further studies are still needed. This review summarises the basic approaches in the fight against the late blight over the past 20 years and includes four sections devoted to methods of control: (1) fungicides; (2) R-gene-based resistance of potato species; (3) RNA interference approaches; (4) other approaches to control *P. infestans*. Based on the latest advances, we have provided a description of the significant advantages and disadvantages of each approach.

## 1. Introduction

For more than 150 years, humankind has been attempting to combat *Phytophthora infestans*. However, despite the wide arsenal of methods involved, most attempts have not been sufficiently effective. Every year, this pathogen causes enormous losses to agriculture worldwide [1]. Fungicides, the most common tool to counteract *P. infestans*, have certain drawbacks: a high price, a prohibited use in organic farming, a potential risk to the ecosystem and health [2], and the opportunity for resistant strains to emerge [3,4]. It is the latter one that is of key importance and somehow contributes to the manifestation of the others.

In the past, cultivation of plant varieties resistant to *P. infestans* was attempted, but it failed due to the pathogen’s adaptation being immeasurably faster than that of the host [1]. With gene-editing technologies evolving and knowledge about the functions of host and parasite genes increasing, this approach is showing renewed promise. Currently, there is an active search for resistance genes (R-gene) that *P. infestans* cannot overcome quickly.

*P. infestans*, like many other pathogenic organisms, produces a variety of effector proteins that can change the host’s physiology, combat its immune response, and facilitate invasion. The recognition of these effectors underlies the R-gene activity, while *P. infestans* has various molecular and genetic mechanisms that allow it to avoid recognition [5,6].

*P. infestans*, known as one of the most aggressive pathogens, has some special features that determine its high adaptability to the host *S. tuberosum.* Compared to other oomycetes, *P. infestans* has a huge genome (240 Mb) with an extraordinary organisation. It consists of blocks with conserved gene sequences with relatively low numbers of repeats, separated by sparse regions with alternating gene sequences, having low density but a large number of repeats [1,2]. The *P. infestans* genome is extremely rich in transposons, occupying about one-third of the total, together with repeats and sparse regions (where the genes are located far from each other). All these factors stimulate mutational variability in *P. infestans* [1]. Rapidly mutating secretory effector genes are mainly located in sparse regions [1].

Given the high rate of *P. infestans* evolution, point measures are inefficient and costly, making systemic countermeasures crucial: combining R-genes [7], selecting the dose and time of fungicide use [8], monitoring populations [9,10], using symbiotic bacteria [11,12] etc. At the same time, fundamentally new RNA-interference-based technologies are being developed, opening up the possibilities of both efficient shutting down of host genes (e.g., immunosuppressors) [13] and creating environmentally-friendly fungicides with an unlimited range of potential targets [14]. In the following sections, each of the methods mentioned is discussed in more detail.

## 2. Methods of Control

### 2.1. Fungicides

Fungicides are the oldest weapon used against late blight. The first of these was the copper-containing “Bordeaux mixture” (also called “Bordo Mix”) used in the 19th century. Nowadays, given the lack of resistant cultivars, fungicides remain the most common method of controlling *P. infestans* [15]. The main advantages of fungicides are their efficiency and simplicity in production and use. According to the action principle, fungicides are divided into the following groups.

- Protective fungicides: effectively prevent infection, but do not help if the plant is already infected;
- Antisporulants: prevent infection from spreading;
- Translaminar fungicides: penetrate leaf blades;
- Curative fungicides: have limited curative effects in the case of active infection;
- Systemic fungicides: can effectively move within the host plant’s vascular system and protect even the new parts of the plant that grow after treatment.

A fungicide is capable of exhibiting different modes of action [16]. Systemic fungicides include metalaxyl and its active R-isomer—mefenoxam, both having a negative impact on *P. infestans* ribosomal RNA synthesis [17].

The most cost-effective and popular control method is complete disease prevention to be achieved through comprehensive measures involving regular (usually weekly) treatment with a mixture of protective and systemic fungicides, for example, mixture of a widely specific and a narrowly specific fungicide. The use of fungicides with a different defence mechanism allows one to resist late blight more comprehensively and, ultimately, reduce the amount of chemicals used. The protection of the tubers from contamination is also important with selecting the combination of active ingredients [18,19,20]. It is a high-cost strategy, and the environmental impact of using large amounts of fungicides remains to be investigated [21]. However, the benefits of such preventive measures, including the difficulty of pathogen adaptation to them, outweigh the costs [18,22,23].

#### 2.1.1. Resistance: Causes and Effects

One of the main problems associated with fungicide use seems to be the resistance subsequently acquired by *P. infestans*. There is a so-called “arms race” in the fight against late blight on all fronts, and fungicides are no exception. Unfortunately, continuous mass application of fungicides causes increased evolutionary pressure on *P. infestans* and consequently may initiate rapid adaptation and acquisition of resistance to a fungicide involved [24]. Field treatment of potato crops with seven different fungicides demonstrated that *P. infestans* could develop resistance to them in one season [25]. If no other control methods are applied, the acquired trait becomes fixed in the population, the resistance increases, and subsequent control requires either a higher initial treatment concentration or using a new fungicide that was not used before, resulting in higher costs.

The induction of ATP-binding cassette transporters (ABC transporters) is one of the factors causing resistance to these chemicals. The resistance develops in response to metalaxyl and appears to be due to epigenetic control, namely chromatin modification, ensuring the rapid development of this trait in a previously sensitive population [15,17]. ABC transporters actively pump metalaxyl and other harmful substances out of the parasite’s cells, thus reducing their impact on the oomycetes. Changes in the parasite’s plasmalemma, preventing poisons from entering its cells, also reduce its sensitivity to metalaxyl [26]. Such protection mechanisms ensure a reduced basic sensitivity to various substances. The sensitivities of different *P. infestans* lineages show ten-fold differences [22]. ABC-transporters and detoxifying enzymes such as cytochrome P450 allow *P. infestans* to survive the “first shock” from an encounter with a new chemical, but, in general, their effect is limited and not very significant. However they do underlie the resistance to mefenoxam/metalaxyl [15,27,28]. Resistance to these resulting from detoxification mechanisms may even develop after the first treatment of a previously sensitive lineage [3]. However, stable lineages have slightly reduced aggressiveness and viability. In the absence of metalaxyl/mefenoxam in the environment, the pathogen loses its acquired resistance to the substance after several generations [17]. Metalaxyl was first used in Europe in 1979, but the following year, *P. infestans* resistance to it was reported. In the USA, methylaxyl continued to be used until 1989, when it became less effective [22,29]. However, by the 2010s, three of the six *P. infestans* lineages common in the United States were found to be sensitive to mefenoxam/metalaxyl, while another lineage had limited sensitivity [22,30]. Thus, in order to maintain the effectiveness of metalaxyl or mefenoxam, it is sufficient to alternate them with other fungicides. The same recommendation is applicable to any fungicide adaptation that results in reduced viability of *P. infestans*.

Fungicides can also be divided into two groups: specific and broad-spectrum fungicides. Broad-spectrum fungicides include sulphur, copper sulphate, other copper compounds popular in organic farming. Mancozeb is also widely used against late blight. Such substances are often more toxic to humans (mancozeb being one of the lucky exceptions) than the narrowly specific ones. However, the development of fungal resistance to them is much less likely [27,31].

Most chemicals used against late blight are site-specific (affecting specific metabolic pathways): azoxtitrobin, fluazinam, mandipropamid, metalaxyl, etc. Their specific toxicity makes them safer for the environment and humans while increasing the risk of *P. infestans* developing resistance through a single mutation, though possibly taking time and leading to reduced viability [22,27,32]. However, such fungicides are still considered sufficiently effective and are widely used, primarily because their correct application reduces the evolutionary pressure of each pesticide on *P. infestans*.

**Figure 1:**
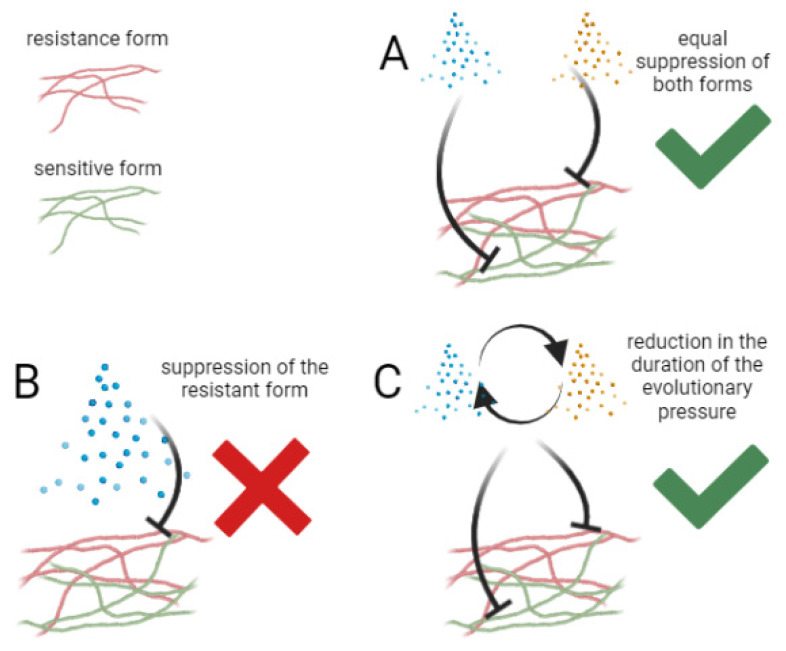
Three strategies for reducing the evolutionary pressure of fungicides on pathogens: (**A**) equal suppression of the growth of both forms; (**B**) suppression of the growth of the resistant forms compared to the sensitive ones; (**C**) and reduction in the duration of the evolutionary pressure.

What is the best way to use fungicides? The development of resistance in a population is defined as an increase in the proportion of resistant forms in relation to the sensitive forms in the population [8]. Based on this, three strategies have been proposed to reduce the evolutionary pressure of fungicides on pathogens: equal suppression of the growth of both forms, suppression of the growth of the resistant forms compared to the sensitive ones, and reduction in the duration of the evolutionary pressure. The second strategy is quite challenging to implement, but the first and third are actively used (Figure 1) [8].

The first strategy requires using a mixture of a widely specific and a narrowly specific fungicide and is the most popular of the three. It is preferable as the development of resistance to both fungicides at once is very unlikely [33]. In the case of *P. infestans*, mancozeb, which has a purely protective effect, is typically applied in association with a systemic fungicide. Another option is to use two narrowly specific fungicides with different modes of action.

The third strategy is somewhat less popular but also demonstrates a reduced rate of resistance development [8]. In practice, it can be implemented by alternating the use of two fungicides with different targets or types of action, thus reducing the exposure time of each fungicide.

#### 2.1.2. Resistance Acquisition and Spread

Monitoring the sensitivity of *P. infestans* regional lineages to the fungicides applied is an important aspect of preventing disease outbreaks and repeated resistance development [4,34,35]. Application of a fungicide to which local lineages are highly tolerant is dangerous for several reasons. The trait may firmly fix in the population, and the population may then spread to other areas or go through a stage of sexual propagation contributing to the fixation of the trait [36]. Additionally, it is worth considering the possibility of an epidemic should such resistance be detected late, as happened in the USA in 1990 [29]. Similarly, the early detection of the lineage’s sensitivity to metalaxyl could have helped prevent the epidemic caused by the US22 lineage in 2009 in the United States [9]. Today, rapid advances in computer technology, meteorology, and molecular biology have allowed us to reach a new level of *P. infestans* observation. Molecular genetic markers have been used to precisely determine the *P. infestans* clonal lineages for quite a long time [10]. The next step should be to analyse both old and new lineages for their resistance to fungicides and even R-genes (see below) and track distribution and recombination. This information should be integrated into available databases such as the Decision Support System (DSS) in the USA [9]. Accurate long-term prediction of weather conditions is one key to saving on fungicide wastage. In years unfavourable for late blight development, fungicide use can be significantly reduced without any risk of crop loss. Such an approach would accumulate resources to control future outbreaks, reduce the evolution pressure on *P. infestans*, and reduce the potential negative impact on the environment [37].

Due to the availability and dissemination of information on fungicides and the pathogens resistant to them, including *P. infestans*, it is worth mentioning the Fungicide Resistance Action Committee (FRAC) aiming to identify resistance development risks, coordinate research on this topic, help in the correct use of fungicides, and classify fungicides [27].

#### 2.1.3. Economics

The economic efficiency of fungicide application is closely related to the global struggle with late blight. Some producers cannot afford the full range of protective procedures, or they use obsolete substances. In such cases, there is a risk that a new lineage of *P. infestans* might develop and migrate to other regions. For example, in Kenya, farmers often cannot afford the integrated application of protective fungicides. Additionally, there are regions where rainy weather may make their application difficult: on the one hand, mancozeb becomes active only in water, and on the other hand, it can be easily washed off the leaves by rains [19]. In regions where small farms predominate or where producers’ incomes are generally low, the fungicide cost is critical for the *P. infestans* control effectiveness. According to ROSSTAT (Federal State Statistics Service of the Russian Federation) [38], private households and farming enterprises in the Russian Federation account for about 85% of all cultivated areas planted with potato crops, making Russian potato plantations particularly vulnerable to late blight.

Mancozeb has been used for over 46 years and remains the most popular broad-spectrum fungicide against *P. infestans* and other fungi. It offers a combination of anti-fungal and economic efficiency with low side toxicity. The agrochemical industry has not yet produced anything to surpass mancozeb. However, with the world’s growing population increasing the demands on agriculture, something new is sure to prove necessary soon [39].

The easiest way to increase the economic efficiency of fungicides is to use them in smaller amounts. Manufacturers often indicate unreasonably high required dosages in their treatment protocols to make additional profit. In various situations, the differences in net income resulting from the use of fungicides range from €167 to €656/ha [18]. The required dosage depends on the sensitivity of both the *P. infestans* and the potato and the weather conditions [16,18,37]. Correct assessment of the infection pressure allows a reduction in the amount of applied fungicide by up to 30% [23]. In some cases, the fungicide can be partially replaced by a cheaper analogue without loss of efficiency [40]. Finally, the use of resistant potato varieties, especially those with field resistance developed due to quantitative trait loci (to be discussed shortly), provides an average increase in net income of about €900/ha, partly due to the significant reduction in the volume of applied fungicides required. All these facts illustrate the importance of developing and introducing new potato varieties resistant to *P. infestans*. Even then, the degree of tuber protection must be carefully monitored in the case of reduced fungicide application [18].

#### 2.1.4. Fungicides in Organic Farming

Fungicides are of particular concern in organic farming where using synthetic substances and, hence, the vast majority of traditional fungicides is prohibited. The most common and effective fungicide approved for organic farming is copper, with its effect related to the reduction in abscisic acids in treated plants [41]. Other substances are much weaker in controlling the late blight spreading. However, copper is inferior to synthetic fungicides and often more toxic. When used frequently, copper can not only accumulate in soil and kill a wide range of soil microorganisms, but it can also cause dermatitis in humans [16]. Thus, it is questionable whether the complete rejection of synthetic chemicals in favour of copper in organic farming is justified. Due to the restrictions on the use of pesticides, organic farming is a sector of agriculture that is particularly vulnerable to *P. infestans* [9,42]. Therefore, organic farms represent a “weak link” even in a region where other producers can afford comprehensive measures to prevent *P. infestans* outbreaks. The same goes for private households.

Thus, since fungicides are the most popular and effective way to protect crops against *P. infestans*, their consumption will only increase as long as there is no commercial alternative. Proper management, development of new agents, and toxicity control are sure to allow minimising the resistance development risks, resistant specimen proportion in the population, and possible negative fungicide impacts on the environment. Systematic and proper use of fungicides is expensive, and not every entrepreneur is able or willing to pay for it. Therefore, providing assistance to small farms, training farmers in fungicide use and control, creating public databases such as the DSS database and international organisations such as FRAC, in other words, globalising the control of *P. infestans*, can make fungicide application as effective as possible. The more global and organised their application, the more likely they are to defeat *P. infestans*.

Modern sequencing technologies, molecular genetic markers, and computer data processing make it possible to track changes in *P. infestans* populations at the genetic level and plan appropriate responses based on these changes. Computer models of *P. infestans* metabolism, similar to those in [43], may be used in the future to create powerful, specific fungicides with a low risk of developing resistance to them. The evolutionary affinity of oomycetes and the *Apicomplexa* is another promising source of ideas for chemical development [44]. In the future, fungicides will have to be improved to become more precise in their action, cheap and effective to meet the growing demands of agriculture. This task will require all the accumulated knowledge on the biology of *P. infestans* and its closest relatives.

#### 2.1.5. Plant Resistance Inducers

The first studies of plant resistance inducers, or PRIs, appeared as early as the 1900s. However, it is only recently that the methods have been developed that can help understand the variety of their mechanisms of action. Broadly speaking, PRIs are the substances that activate the plant defense mechanisms, for example, by enhancing phytoalexin production and NO reduction in sterol production or by triggering a hypersensitive response [45]. There are numerous combinations, and only some have a known true mechanism.

The main advantages of PRIs are their safety, as they stimulate plant defense rather than poison it, low cost, and broad-spectrum and systemic activity. However, the effect of applying PRIs alone is not sufficient to provide complete protection [46] and may negatively affect plant growth and development [47]. For example, β-aminobutyric acid (BABA) is known to cause necrotic lesions on potato leaves under treatment, due to a local hypersensitive response activation [48]. In each case, the fitness cost depends on the particular PRI, the growth and application conditions.

Taken together, the factors mentioned above make PRIs an ideal adjuvant that is actively used to counteract *P. infestans* [49,50,51], including applications in developing countries [52]. Combining PRIs with fungicides has the best effect by simultaneously increasing the protection and reducing the risk of resistance development while reducing the environmental effects (by minimizing the amount of fungicide used) [40,53]. In fact, it is possible to achieve the first strategy of using fungicides (see above) by substituting one fungicide with a PRI.

Due to their versatile effect and low cost [52], phosphites are frequently used as PRIs. Studies show significant transcriptome [54] and proteome [55] changes after treatment of potato plants with phosphites, leading to reduced phytophthora damage. A uniquely significant feature of phosphites is their suitability for tuber protection [56]. Among other PRIs used against *P. infestans*, it is worth considering the above-mentioned BABA, which is not inferior to phosphites in popularity and breadth of effect. Novel substances are being actively developed. For example, bis-aryl-methanone compound NUBS-4190 triggers NO synthesis without activating the hypersensitive response [51].

Using PRIs proves to be a good alternative to the mass application of fungicides, an opportunity to solve the controversy with organic farming, and to reduce the economic and ecological burden on society. The PRIs perfectly suit the concept of comprehensive counteraction against *P. infestans*.

### 2.2. Genetic Resistance: Avr vs. R-genes

As early as the last century, Harold Henry Flor showed that the inheritance of both resistance in the host and the parasite’s ability to cause disease is controlled by pairs of matching genes [57]. One type is a plant gene called the resistance gene (R-gene). The other type is a pathogen gene called the avirulence gene (Avr-gene).

#### 2.2.1. Variety of Genes in *P. infestans*

A large number of cytoplasmic effectors of *P. infestans* depend on Avr-genes. A great majority of them are the so-called RXLR effectors containing an N-terminal (amino-terminal) motif Arg-X-Leu-Arg, with X being any amino acid. This motif determines the domain required for delivery into plant cells. In addition to the N-terminal conserved domain, RXLR effectors have a large variety of domains in the C-terminal region, specifically in the region required for their effector function and recognition by the plant R-genes [58,59]. *P. infestans* has over 550 RXLR genes [60].

There is also another group of Avr-effectors—the Crinkler (CRN) group. CRNs are defined by a rather conserved N-terminal 50-amino acid domain, the LFLAK, and a related diverse domain DWL. This is also a huge family of approximately 200 genes, and in *P. infestans*, there are also about 250 disrupted and fragmented CRN genes [60].

Both RXLR and CRN are modular proteins mainly located in the gene-sparse regions, with a large number of transposons, repeats, and mobile elements, resulting in their faster mutation rate [24,60].

#### 2.2.2. Variety of Plant R-genes

The molecular structure of R-genes consists of a group with two conserved domains: a nucleotide-binding site (NBS) and a leucine-rich repeat (LRR) domain [61].

R-genes against *P. infestans* (Rpi-genes) are easier to introduce than quantitative trait loci [62] (to be discussed in Section 2.2.6). For this reason, they are being actively studied to create late blight resistant varieties.

In the middle of the 20th century, eleven resistance genes R1 to R11 were found in wild *Solanum demissum* (hexaploid) [41,63,64]. At the beginning of the 21st century, R-gene sources were also found in other wild species in Central America, where *P. infestans* originates from. Four loci with so-called ‘quantitative’ resistance to late blight have also been found in *Solanum bulbocastanum*: Rpi-blb1/RB [65], Rpi-blb2 [66], Rpi -blb3 [67] and Rpi-apbt [68]. The quantitative resistance mechanism does not completely block the late blight infection but slows down the disease progression, reducing the damage caused. R-gene sources have also been found in tuberous species of the *Petota* section originating mainly from North, Central, and South America [62], and it is thought that there are possibly R-gene sources in other wild species of *Solanum*. Thus, more than 20 genes of quantitative resistance to *P. infestans* have been discovered, with all of them having an N-terminal motif containing two loops (NB-LRR) [66,69,70,71,72,73,74].

However, genes of specific resistance, i.e., qualitative ones (with the plant completely resistant to the pathogen), may also be of interest. Thus, the R8 gene has been found in late blight resistant potato varieties from Europe (Sarpo Mira), the USA (Jacqueline Lee, Missaukee) and China (PB-06 and S-60). The R8 gene recognises Avr8 and is homologous to the Sw-5 NB-LRR protein of tomatoes. In field trials in the Netherlands [74], transgenic potatoes with R8 demonstrated a wide range of resistance to the current population of *P. infestans*. Later it was also demonstrated that the resistance of potatoes with locus dPI09c on chromosome 9 (which is a reasonably strong source of field resistance against *P. infestans*) could be explained by the presence of the R8 gene [75]. It was the first time when a gene of supposedly limited resistance demonstrated a broad spectrum of effect and long-term field resistance. Therefore, additional research on the R-genes that provide limited resistance may be promising.

#### 2.2.3. Interaction of R- and Avr-Effectors

Immune receptors can detect Avr-effectors directly via protein-protein interactions or indirectly by the detection of host target modifications or host mimicry [24]. Interaction of the corresponding R-genes and the RXLR avirulence genes causes a hypersensitive response (HR)—a localised programmed death of any cells infected with *P. infestans* [24].

It was suggested that cultivated varieties obtained by selective breeding with wild resistant plants that have appropriate R-genes are among the most effective, environmentally friendly and cost-effective methods of controlling *P. infestans* [62]. Resistant varieties should contain R-genes that are able to recognise the corresponding Avr-genes and thus cause a hypersensitive response (HR) [24]. Unfortunately, some resistant varieties were found to be defeated in just one season because the resistance genes targets—the RXLR effector genes—evolve very rapidly through gene insertions and deletions, complete gene deletions, point mutations (SNPs), present and absent variation (PAV), and gene silencing, avoiding interactions with the R-genes [76,77]. The evolution of RXLR genes is also facilitated due to the vast majority of them being located in sparse regions with a large number of transposons, mobile elements, and repeats [1,2,24,60]. Thus, co-expression of the Avr1 gene with R1 leads to a hypersensitive response in *Nicotiana benthamiana* plants, whereas such an effect does not occur for its homolog A-L [78].

#### 2.2.4. New Data in Understanding R-gene Function

Conserved effector genes of *P. infestans* expressed at an early stage of invasion suppress the host’s immune response. Thus, high expression of the SFI2, SFI3 and SFI4 Avr-genes (suppressors of the Flg22-induced immune response) in five strains has demonstrated that their function in the early stages of invasion could be significant [79]. The analysis of 10 Avr-genes allowed predicting that these genes could provide long-term resistance.

It is important to note that different R-genes can provide resistance to the same Avr-gene. Thus, the R2 gene of a Mexican species and the Rpi-mcq1 of a Peruvian species of *Solanum* are sensitive to the Avr2 gene [80]. It is claimed that Avr2 recognition developed independently at the two genetic loci.

Despite numerous studies on R-gene mapping in the potato genome [69,70,81,82,83,84,85,86,87], it should be noted that resistance to the R-gene pathogen in potato varieties persists for 5–10 years, and then the variety becomes susceptible to new races of *P. infestans* [88]. Pathogen recognition by the R-gene is fairly rapidly mitigated by mutations in the corresponding *P. infestans* avirulence gene, allowing the pathogen to successfully penetrate and colonise the host plant in a compatible interaction [89].

In addition to single dominant R-resistance genes responsible for recognising the corresponding *P. infestans* avirulence gene and triggering a defence response manifested in local cell death (hypersensitivity reaction) and thereby stopping the growth of pathogenic microorganisms, there is a group of genes with another defence mechanism—plants’ multiple resistance genes. The expression of four transporter genes in potatoes, with transcription regulated by different drugs, was examined [90]. Among those, other genes were found with a significant expression increase upon infection with *P. infestans*: StPDR1 and StPDR2 were expressed 13- and 37-fold more actively after 18 h of infection, respectively. The authors suggested all the genes studied (StPDR1-4) to be part of a more complex systemic plant response to biotic and abiotic factors.

#### 2.2.5. Gene Pyramids Provide a Boost to R-genes

It was suggested that the Rpi-blb1 and Rpi-blb2 genes obtained from *S. bulbocastanum* could be a key to long-term resistance [21]. It should be noted that *P. infestans* subsequently developed resistance to Rpi-blb1, although it took longer than the adaptation to previously used R-genes [15]. However, the *S. bulbocastanum* Rpi-blb2 gene combined with the *S. venturii* Rpi-vnt1.1 gene did make potatoes fully resistant to late blight for several seasons [91]. This phenomenon is referred to as the gene pyramid. Here, multiple genes control a trait, such as resistance to pathogens, and accumulate and combine into a single genotype [7]. However, positive selection of the Avrblb2 gene (related to the RXLR genes) is seen to be underway in *P. infestans* populations. At least four variations of this gene have emerged, with one of them evading the Rpi-blb2-related gene [92]. This was found to result from a mutation and replacement with phenylalanine in position 69 [17]. From this perspective, it is probably a combination of several R-genes that will provide potatoes with the longest-lasting resistance. Specifically, the Rpi-blb1 and Rpi-blb2 genes are considered to be very promising. *P. infestans* rarely demonstrates resistance to them.

Another example of the R-gene combination providing long-term effectiveness is the Cooperation-88 (C88) potato variety that has been demonstrating high resistance to *P. infestans* for over 20 years. This resistance was found to be provided by 344 expressed R-genes, as well as nine genes associated with pathogenesis, and a sharp increase in the expression of 30 genes responsible for phenol compound synthesis in case of invasion, showing that R-genes need phenol compounds and pathogenesis proteins to provide excellent resistance to late blight [93].

Thus, the *P. infestans* genome is well adapted to overcome recognition by R-genes, and all attempts to develop a variety with long-lasting resistance based on selective breeding or methods of genetic engineering of just a single R-gene have failed. However, recent studies of *P. infestans* Avr-genes show that conservative genes are present among them and that the genes Avrpi-blb1 and Avrpi-blb2 are quite conservative. Moreover, *P. infestans* strains capable of overcoming them are not yet common. Further research on conservative Avr-genes and the pyramiding of resistant R-genes could give a boost to qualitative resistance in potatoes.

#### 2.2.6. Quantitative Trait Loci (QTLs)

QTLs (those affecting height and weight) involve sets of alleles affecting a trait with a measurable phenotypic value resulting from both genetic and environmental factors. Quantitative traits are typically multifactorial and are controlled by the interaction of several polymorphic genes and environmental factors. Therefore, one or more QTLs may affect one trait or the entire phenotype. QTLs cannot be set in opposition to so-called Mendelian loci, functioning on the “all or nothing” principle. These are rather the two extremes of the same line, with QTLs occupying one pole and discrete Mendelian loci occupying the other [94].

Thus, two regions of chromosomes V and XII of *S. tuberosum* matched with the dominant allele of the R1 gene that gives specific resistance to late blight [95]. The main problem in finding and mapping QTLs for *S. tuberosum* is its wide genetic diversity [96]—its autoploid has tetrasomic inheritance and a high degree of heterosis [97]. Thus, the potato genome has great structural complexity, constraining the analysis of quantitative trait loci [98]. However, six QTLs of resistance to *P. infestans* have been found: two QTLs of sensitivity and four QTLs of resistance [97]. The analysis of different QTLs shows that it is the R-genes that tend to be responsible for resistance to late blight. The resistance of potatoes with locus dPI09c on chromosome 9 was conditioned by the presence of R8 [75]. The efficacy of the promising Rpi-blb1 gene also appears to be dependent on the genome’s genetic background, and therefore seems to be related to QTLs [99]. Furthermore, the variety 3681ad1 that shows good field resistance (each plant is individually vulnerable, but the field as a whole is resistant) turns out to have a dominant allele of the R10 gene in one of its QTLs [100].

A comprehensive attempt to map potato quantitative trait loci was undertaken in 2018 [97]. The tetraploid potato genome was again chosen as a research target, given its high importance for breeding and yet significant challenges due to high heterozygosity in autotetraploid potatoes. The researchers succeeded in discovering two new QTLs on chromosomes III and VIII. One allele of the first locus was reported to mediate, on average, a higher degree of disease severity. This locus also includes the transcription factor Arf 2 associated with leaf senescence caused by oxidative stress in Arabidopsis and gibberellin and brassinosteroid pathway signalling during plant-pathogen interaction [101,102,103]. The allele determining, on average, a lower disease severity contained the QTL of chromosome VIII. This marker is related to the gene encoding the helix-loop-helix transcription factor (bHLH) JAF13 involved in flavonoid biosynthesis in Petunia × hybrida [104].

QTL functioning principles are not yet fully understood and difficult to detect as well. Nevertheless, the study of quantitative resistance loci is underway and seems to be promising. The recent interest in QTLs has been caused by *P. infestans* quickly adapting to become resistant to the potato’s own R-genes, so the application of QTLs seems to be promising for developing varieties with quantitative resistance.

#### 2.2.7. Application of Resistant Varieties

While producing resistant potato varieties, we are entering an arms race. In nature, there is constant joint evolution of the *P. infestans* Avr-genes and the potato R-genes. Potato R-genes constantly transform in order to identify *P. infestans* Avr-genes to be able to cause local programmed cell death. It is an effective strategy because *P. infestans* is, exceptionally, a biotroph, not a necrotroph. However, the Avr-genes themselves rapidly evolve, or their function is altered to avoid interaction with R-genes, preventing the plant from triggering a hypersensitive response.

The increasing spread of sexual propagation and self-fertilisation among *P. infestans* strains also speaks against the application of R-genes. Thus, there is a positive correlation between the complexity of the Avr-genes set and the viability, whereas, normally, there should be no such correlation [36].

Nevertheless, the development of resistant varieties is being actively pursued worldwide, both by traditional selective breeding and genetic engineering. Selective breeding to develop a resistant variety takes longer than creating a transgenic plant. However, in many countries, the use of transgenic organisms is prohibited or severely restricted, making it challenging to introduce new, resistant varieties quickly. Currently, the most promising approach is to develop varieties with a combination of several R-genes, such as Rpi-blb1, Rpi-blb2 and their homologs because they target fairly conserved and, therefore, important *P. infestans* Avr-genes. Although some strains of *P. infestans* are now resistant to these genes, such clonal lineages are not very common. Using the gene pyramid of Rpi-blb1 and Rpi-blb2 with other genes of quantitative and qualitative resistance should allow creating a variety that would be resistant for more than a decade, such as the Cooperation-88 variety that has retained resistance for more than 20 years. Further research on QTLs and analysis of the genes that make up these loci may also be promising in finding a wider diversity of resistance genes.

### 2.3. Use of RNA Interference against P. infestans

Plant immune systems are complex. There are still numerous uncertainties in their functioning, but many separate mechanisms have been studied in reasonable detail. One of them is the expression of small RNAs (sRNAs) that act as silencers of the pathogen genes responsible for virulence [24]. The use of RNA interference by plants for protection against fungi (including oomycetes) has a number of features that distinguish it from antiviral or antibacterial protection. As fungi have their own RNA interference pathways, in fact, we are dealing with “cross-kingdom RNA interference” [105]. The presence of similar RNA interference pathways both in the parasite and host led to the development of control interception methods in both plants and fungi. Fungi and oomycetes can directly disrupt the RNA interference function in plants [106] or assign it to decrease the host immunity [107]. Plants use extracellular vesicles to deliver small RNA agents or their precursors to the pathogen [108]. The use of small RNA transfer from host plants to the pathogen with subsequent intervention in its RNA interference pathways has been termed host induced gene silencing (HIGS). In recent years, the practical application of this technology has been studied extensively, including its application against *P. infestans* [105,109,110]. This section includes an overview of the current RNA-interference-based methods of controlling various pathogenic fungi in plants that can be used against *P. infestans.*

#### 2.3.1. HIGS: Prospects and Challenges

**Figure 2:**
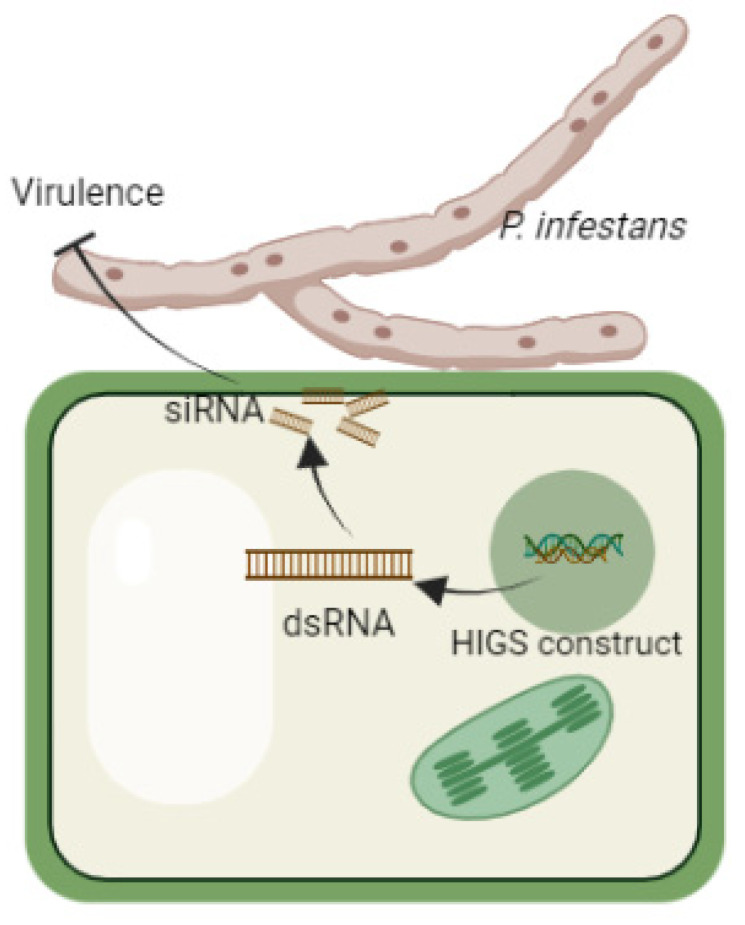
The scheme of HIGS action against *P. infestans*.

First of all, it is worth noting the direct use of HIGS, i.e., the creation of transgenic plants that express predetermined siRNA, dsRNA or hpRNA, that can cause knockdown of targeted vital genes of the pathogen (Figure 2) [111,112,113]. A correct choice of the target gene (or several target genes) is crucial [112]. There are currently several commercial varieties in the USA in which HIGS is effectively used to fight viruses and insects, but none yet combat oomycetes [114]. As a matter of interest, some of them were obtained before the discovery of RNA interference in the 90s, and proteins were originally considered to be responsible for the resistance [114,115]. The use of RNA interference in these varieties is a lucky coincidence, while other commercial varieties using HIGS appeared about ten years later [114]. It seems strange that HIGS varieties have not subsequently flooded the market because a long time has passed since the effectiveness of RNA interference was demonstrated. This technology has been sufficiently well-researched in laboratories around the world [116] to create plants with advancements based on RNA interference. However, the issue is not with the RNA-interference itself but with the methods of its application. HIGS requires, in the first place, creating a transgenic plant, not a single specimen but a whole variety. The development of GMOs is justified by the market when it comes to improving something permanent: crop yields, nutrient value, etc. However, investing in creating a pathogen-resistant variety means becoming involved in an arms race with sometimes unclear results and long-term benefits. There are few transgenic varieties on the market with resistance to pathogens because, as a rule, this risk is justified only when the crop’s economic importance is matched by effective and lasting resistance [117].

Introducing a new variety to the market also includes growing planting material and testing its long-term resistance, and it is here that issues may arise. For example, the potato is tetraploid and has a high degree of heterozygosity, demonstrating significant variability of traits even after the same treatment with modern methods of genetic engineering [21,37]. Moreover, as a rule, field experiments show different results from those found in the laboratory. *P. infestans* cultivated on agar or in a potato field behaves differently (the laboratory lineages are less aggressive), which can cast doubt on laboratory evaluation of the research results [37].

One of the most significant limitations on developing the commercial late blight-resistant potato varieties is the availability of effective fungicides: fields are planted with vulnerable varieties and are treated with huge amounts of such chemicals [21]. In recent years, the USA has consumed up to 5 million pounds of fungicides per year to fight *P. infestans* alone [15]. Thus, it is challenging not only to develop a new variety but also to introduce it to a busy market. Moreover, although there may be enough time to choose a fungicide against a particular strain, a new plant variety must be chosen in advance—before planting. Another issue related to using transgenic plants is the legal restrictions that either completely prohibit or effectively inhibit the production of genetically modified products [118]. These factors apply not only to HIGS plants but also to transgenic plants and new varieties in general. When creating a variety resistant to *P. infestans,* researchers may face greater economic and social challenges than scientific ones [117]. One way to solve this problem more easily is not to change the plant genome itself, if possible, but to use external interferences, and RNA interference is a case in point.

#### 2.3.2. Spray Induced Gene Silencing (SIGS)

**Figure 3:**
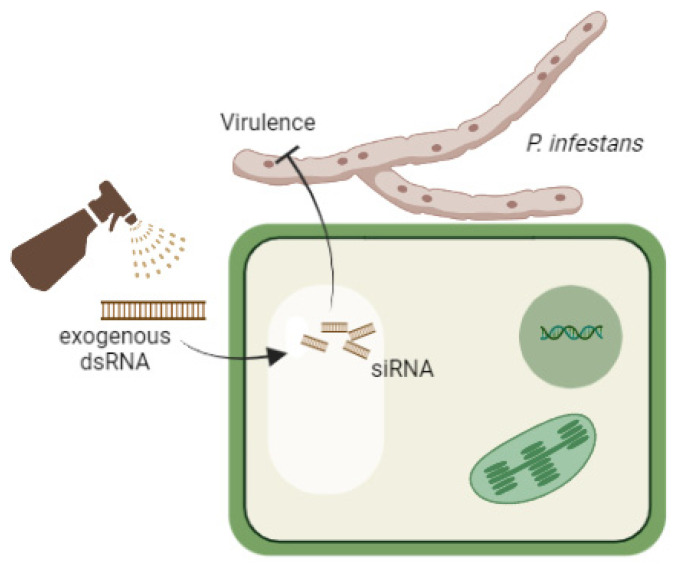
The scheme of SIGS action against *P. infestans*.

SIGS is an alternative option for pest control [103] by RNA delivery into the plant from outside through the leaves and roots. The technology involves an aqueous solution of sRNA, hpRNA, or even siRNA created in vitro or produced by bacteria, applied by spraying plant leaf surfaces, inoculation, injection or through the root system (Figure 3) [110]. The effect of exogenous RNA on plant metabolism was reported [119] and parasitic fungi such as representatives of the *Fusarium* genus were characterised [120]. The work [120] describes the route of RNA directly to fungus through the plant. The silencing duration is related to the secondary amplification of the siRNA, but such amplification does not occur in the fungus. Thus, it makes sense to find out how *P. infestans* interacts with directly received RNA when adapting SIGS to act against *P. infestans*. For example, despite the fact that *P. infestans* has well-developed RNA interference pathways that include siRNA and miRNA, it obtains a significant proportion of small RNA (sRNA) from pre-tRNA without DICER-like proteins (PiDcl1), with the presence of sRNA increasing during infection [121]. Interfering RNAs are of particular importance for oomycetes in the early stages of infection since they take part in knockdown of the pathogen’s “uncovered” gene-effectors that allow the pathogen to overcome the resistance caused by R-genes [24].

SIGS is drawing the researchers’ attention as a promising approach to create biofungicides that combine the efficiency and nontoxicity of HIGS with the ease of use of chemical fungicides [14]. Unlike HIGS, SIGS can be used to protect an already harvested crop [14], and this can be crucial in combating *P. infestans* that can travel long distances on tubers being transported, and causes great damage to them [15] during their storage [21]. Exogenous use of RNA is a prospective strategy, the future effectiveness of which depends not only on fundamental understanding of its action mechanisms but also on experimentation with its use [110]. One can already expect the parity in the cost of conventional fungicides and exogenous dsRNA to be reached soon [64], but application strategies may need to be refined. The dsRNA application efficiency varies considerably among fungal and oomycete species, and data on *P. infestans* are still scarce [65].

### 2.4. Other Counter P. infestans Approaches

There are various other easily applicable methods for preventing late blight outbreaks. Among the most important are crop rotation and the elimination of primary sources of infection [122]. In regions with mild winters, tubers infected with *P. infestans* and left in the field during harvesting may infect newly planted potato/tomato plants the following year [9]. Alternating potatoes with other crops immune to late blight every other year is helpful in avoiding recurrence of the disease. *P. infestans* spores, however, may remain viable even after a two-year interval. Thus, if sexual propagation is possible in the harvesting area, planting intervals of vulnerable crops should be increased. Planting potatoes remotely from each other also has a small impact on the severity of a possible epidemic [122]. After a long resting period, the viability and pathogenicity of spores decrease significantly [123]. *P. infestans* major route of spreading over long distances is on contaminated planting material, especially tubers. *P. infestans* can survive on tubers in the intercrop season without sexual propagation [28]. Fortunately, only a small proportion of infected tubers can germinate and give rise to an epidemic. However, this can sometimes be enough, given that not all infected tubers/tomatoes in storehouses may be destroyed or cured, and so their transport continues to spread epidemics, as happened in 2014 in Bengal for example [9,37]. Symbiotic bacteria can be used to protect tubers more effectively against infestation [11,12].

Given that it is impossible to eliminate contaminated planting material completely, it is necessary to monitor the state of plants in the field carefully, especially in conditions favourable for the development of an epidemic. Such measures are certainly of auxiliary nature and cannot compete in effectiveness with the use of fungicides or resistant varieties, but their use, especially in organic farming, contributes to the development of a comprehensive response to the threat of late blight.

## 3. Conclusions

*P. infestans* still causes significant damage to agriculture despite the long history of fighting it. A number of physiological and genetic features allow the pathogen to adapt quickly to new control strategies applied by farmers: the emergence of each new method of plant protection starts a kind of countdown to the moment when the pathogen successfully bypasses another obstacle. In order to find new vulnerabilities and develop appropriate strategies, it is necessary to take into account several peculiar features of *P. infestans*, with the main ones described in this review article.

In agriculture, there are several strategies for combating *P. infestans* that can be roughly divided into three categories: chemical (fungicides), genetic (R-gene selection) and gene silencing (a modern approach based on RNA-interference). With each strategy having advantages and disadvantages, one has to take into account the effectiveness of the method, its scalability, environmental feasibility, and environmental safety. Currently, the most common method is the use of various fungicides, which is facilitated by their at-times low but still considerable efficacy, availability and ease of use in plant treatment, as well as the related economic benefits. Selective breeding with resistant varieties is also actively used for crop protection, but *P. infestans* strain prediction must be carefully considered when applying this strategy as prediction errors can be costly. The most advanced and selective methods aimed at precise regulation of gene function with the help of RNA interference have not yet seen wide application in agriculture due to their high cost and legal prohibitions. However, they are very promising from an ecological point of view due to their high degree of selectivity and, as a result, environmental safety.

## References

[1] Haverkort A.J., Struik P.C., Visser R.G.F., Jacobsen E. (2009). Applied biotechnology to combat late blight in potato caused by *Phytophthora infestans*. Potato Res..

[2] Pacilly F.C.A., Groot J.C.J., Hofstede G.J., Schaap B.F., van Bueren E.T.L. (2016). Analysing potato late blight control as a social-ecological system using fuzzy cognitive mapping. Agron. Sustain. Dev..

[3] González-Tobón J., Childers R., Olave C., Regnier M., Rodríguez-Jaramillo A., Fry W., Restrepo S., Danies G. (2019). Is the Phenomenon of Mefenoxam-Acquired Resistance in *Phytophthora infestans* Universal?. Plant Dis..

[4] Schepers H.T.A.M., Kessel G.J.T., Lucca F., Förch M.G., van den Bosch G.B.M., Topper C.G., Evenhuis A. (2018). Reduced efficacy of fluazinam against *Phytophthora infestans* in the Netherlands. Eur. J. Plant Pathol..

[5] Yang L.-N., Liu H., Duan G.-H., Huang Y.-M., Liu S., Fang Z.-G., Wu E.-J., Shang L., Zhan J. (2020). The *Phytophthora infestans* AVR2 Effector Escapes R2 Recognition Through Effector Disordering. Mol. Plant-Microbe Interact..

[6] Du Y., Chen X., Guo Y., Zhang X., Zhang H., Li F., Huang G., Meng Y., Shan W. (2021). *Phytophthora infestans* RXLR effector PITG20303 targets a potato MKK1 protein to suppress plant immunity. New Phytol..

[7] Simko I., Jansky S., Stephenson S., Spooner D. (2007). Genetics of Resistance to Pests and Disease. Potato Biol. Biotechnol. Adv. Perspect..

[8] van den Bosch F., Fraaije B., Oliver R., van den Berg F., Paveley N. (2015). The Use of Mathematical Models to Guide Fungicide Resistance Management Decisions. Fungicide Resistance in Plant Pathogens.

[9] Fry W.E., McGrath M.T., Seaman A., Zitter T.A., McLeod A., Danies G., Small I.M., Myers K., Everts K., Gevens A.J. (2013). The 2009 late blight pandemic in the eastern United States-Causes and results. Plant Dis..

[10] Lees A.K., Wattier R., Shaw D.S., Sullivan L., Williams N.A., Cooke D.E.L. (2006). Novel microsatellite markers for the analysis of *Phytophthora infestans* populations. Plant Pathol..

[11] Yan H., Qiu Y., Yang S., Wang Y., Wang K., Jiang L., Wang H. (2021). Antagonistic Activity of *Bacillus velezensis* SDTB038 against Phytophthora infestans in Potato. Plant Dis..

[12] Wang Y., Liang J., Zhang C., Wang L., Gao W., Jiang J. (2020). *Bacillus megaterium* WL-3 Lipopeptides Collaborate Against *Phytophthora infestans* to Control Potato Late Blight and Promote Potato Plant Growth. Front. Microbiol..

[13] Sun K., Wolters A.-M.A., Vossen J.H., Rouwet M.E., Loonen A.E.H.M., Jacobsen E., Visser R.G.F., Bai Y. (2016). Silencing of six susceptibility genes results in potato late blight resistance. Transgenic Res..

[14] Islam M.T., Sherif S.M. (2020). RNAi-based biofungicides as a promising next-generation strategy for controlling devastating gray mold diseases. Int. J. Mol. Sci..

[15] Leesutthiphonchai W., Vu A.L., Ah-Fong A.M.V., Judelson H.S. (2018). How does *Phytophthora infestans* evade control efforts? Modern insight into the late blight disease. Phytopathology.

[16] Seidl Johnson A.C., Jordan S.A., Gevens A.J. (2015). Efficacy of organic and conventional fungicides and impact of application timing on control of tomato late blight caused by US-22, US-23, and US-24 isolates of *Phytophthora infestans*. Plant Dis..

[17] Childers R., Danies G., Myers K., Fei Z., Small I.M., Fry W.E. (2015). Acquired resistance to mefenoxam in sensitive isolates of *Phytophthora infestans*. Phytopathology.

[18] Wiik L., Rosenqvist H., Liljeroth E. (2018). Study on Biological and Economic Considerations in the Control of Potato Late Blight and Potato Tuber Blight. J. Hortic..

[19] Muchiri F.N., Narla R.D., Olanya O.M., Nyankanga R.O., Ariga E.S. (2009). Efficacy of fungicide mixtures for the management of *Phytophthora infestans* (US-1) on potato. Phytoprotection.

[20] Evenhuis A., Bain R., Hausladen H., Nielsen B.J., van den Berg W., Schepers H.T.A.M. (2019). Fungicide Evaluation to Rate Efficacy to Control Leaf Late Blight for the EuroBlight Table.

[21] Fry W. (2008). *Phytophthora infestans*: The plant (and R gene) destroyer. Mol. Plant Pathol..

[22] Saville A., Graham K., Grünwald N.J., Myers K., Fry W.E., Ristaino J.B. (2015). Fungicide Sensitivity of U.S. Genotypes of *Phytophthora infestans* to Six Oomycete-Targeted Compounds. Plant Dis..

[23] Nielsen, Hansen L., Jens G., Schepers H.T.A.M. (2010). Control of potato late blight using a dose model to adjust fungicide input according to infection risk. Proceedings of the Twelfth EuroBlight, Arras, France, 3–6 May 2010.

[24] Wang Y., Tyler B.M., Wang Y. (2019). Defense and Counterdefense during Plant-Pathogenic Oomycete Infection. Annu. Rev. Microbiol..

[25] Grünwald N.J., Sturbaum A.K., Montes G.R., Serrano E.G., Lozoya-Saldaña H., Fry W.E. (2006). Selection for fungicide resistance within a growing season in field populations of *Phytophthora infestans* at the center of origin. Phytopathology.

[26] Maridueña-Zavala M.G., Freire-Peñaherrera A., Cevallos-Cevallos J.M., Peralta E.L. (2017). GC-MS metabolite profiling of *Phytophthora infestans* resistant to metalaxyl. Eur. J. Plant Pathol..

[27] Kuck K.H., Leadbeater A., Gisi U. (2012). FRAC Mode of Action Classification and Resistance Risk of Fungicides. Modern Crop Protection Compounds.

[28] Fry W.E., Birch P.R.J., Judelson H.S., Grünwald N.J., Danies G., Everts K.L., Gevens A.J., Gugino B.K., Johnson D.A., Johnson S.B. (2015). Five reasons to consider phytophthora infestans a reemerging pathogen. Phytopathology.

[29] Goodwin S.B., Smart C.D., Sandrock R.W., Deahl K.L., Punja Z.K., Fry W.E. (1998). Genetic change within populations of Phytophthora infestans in the United States and Canada during 1994 to 1996: Role of migration and recombination. Phytopathology.

[30] Hu C.H., Perez F.G., Donahoo R., McLeod A., Myers K., Ivors K., Secor G., Roberts P.D., Deahl K.L., Fry W.E. (2012). Recent genotypes of *Phytophthora infestans* in the eastern United States reveal clonal populations and reappearance of mefenoxam sensitivity. Plant Dis..

[31] FRAC (2020). FRAC Code List © 2020: Fungal Control Agents Sorted by Cross Resistance Pattern and Mode of Action.

[32] Gisi U., Walder F., Resheat-Eini Z., Edel D., Sierotzki H. (2011). Changes of Genotype, Sensitivity and Aggressiveness in *Phytophthora infestans* Isolates Collected in European Countries in 1997, 2006 and 2007. J. Phytopathol..

[33] Ojiambo P.S., Paul P.A., Holmes G.J. (2010). A quantitative review of fungicide efficacy for managing downy mildew in cucurbits. Phytopathology.

[34] Zhu G.N., Huang F.X., Feng L.X., Qin B.X., Yang Y.H., Chen Y.H., Lu X.H. (2008). Sensitivities of *Phytophthora infestans* to Metalaxyl, Cymoxanil, and Dimethomorph. Agric. Sci. China.

[35] Rekanović E., Potočnik I., Milijašević-Marčić S., Stepanović M., Todorović B., Mihajlović M. (2012). Toxicity of metalaxyl, azoxystrobin, dimethomorph, cymoxanil, zoxamide and mancozeb to *Phytophthora infestans* isolates from Serbia. J. Environ. Sci. Health-Part B Pestic. Food Contam. Agric. Wastes.

[36] Zhu W., Shen L.L., Fang Z.G., Yang L.N., Zhang J.F., Sun D.L., Zhan J. (2016). Increased frequency of self-fertile isolates in *Phytophthora infestans* may attribute to their higher fitness relative to the A1 isolates. Sci. Rep..

[37] Fry W.E. (2016). *Phytophthora infestans*: New Tools (and Old Ones) Lead to New Understanding and Precision Management. Annu. Rev. Phytopathol..

[38] EMISS. https://www.fedstat.ru/.

[39] Gullino M.L., Tinivella F., Garibaldi A., Kemmitt G.M., Bacci L., Sheppard B. (2010). Mancozeb: Past, present, and future. Plant Dis..

[40] Liljeroth E., Lankinen Å., Wiik L., Burra D.D., Alexandersson E., Andreasson E. (2016). Potassium phosphite combined with reduced doses of fungicides provides efficient protection against potato late blight in large-scale field trials. Crop Prot..

[41] Black W. (1951). XVII—Inheritance of Resistance to Blight (*Phytophthora infestans*) in Potatoes: Inter-Relationships of Genes and Strains. Proc. R. Soc. Edinburgh. Sect. B Biol..

[42] Brazinskiene V., Asakaviciute R., Miezeliene A., Alencikiene G., Ivanauskas L., Jakstas V., Viskelis P., Razukas A. (2014). Effect of farming systems on the yield, quality parameters and sensory properties of conventionally and organically grown potato (*Solanum tuberosum* L.) tubers. Food Chem..

[43] Rodenburg S.Y.A., Seidl M.F., de Ridder D., Govers F. (2018). Genome-wide characterization of *Phytophthora infestans* metabolism: A systems biology approach. Mol. Plant Pathol..

[44] Garavito M.F., Narvaez-Ortiz H.Y., Pulido D.C., Löffler M., Judelson H.S., Restrepo S., Zimmermann B.H. (2019). *Phytophthora infestans* dihydroorotate dehydrogenase is a potential target for chemical control—A comparison with the enzyme from solanum tuberosum. Front. Microbiol..

[45] Eschen-Lippold L., Altmann S., Rosahl S. (2010). dl-β-Aminobutyric Acid–Induced Resistance of Potato Against *Phytophthora infestans* Requires Salicylic Acid but Not Oxylipins. Mol. Plant-Microbe Interact..

[46] Walters D.R., Fountaine J.M. (2009). Practical application of induced resistance to plant diseases: An appraisal of effectiveness under field conditions. J. Agric. Sci..

[47] Walters D., Heil M. (2007). Costs and trade-offs associated with induced resistance. Physiol. Mol. Plant Pathol..

[48] Bengtsson T., Holefors A., Witzell J., Andreasson E., Liljeroth E. (2014). Activation of defence responses to *Phytophthora infestans* in potato by BABA. Plant Pathol..

[49] Fontanilla M., Montes M., De Prado R. (2005). Effects of the foliar-applied protein “Harpin(Ea)” (messenger) on tomatoes infected with *Phytophthora infestans*. Commun. Agric. Appl. Biol. Sci..

[50] Li J., Zhu L., Lu G., Zhan X.B., Lin C.C., Zheng Z.Y. (2014). Curdlan β-1,3-glucooligosaccharides induce the defense responses against *Phytophthora infestans* infection of potato (*Solanum tuberosum* L. cv. McCain G1) leaf cells. PLoS ONE.

[51] Monjil M.S., Shibata Y., Takemoto D., Kawakita K. (2013). Bis-aryl methanone compound is a candidate of nitric oxide producing elicitor and induces resistance in *Nicotiana benthamiana* against *Phytophthora infestans*. Nitric Oxide.

[52] Kromann P., Pérez W.G., Taipe A., Schulte-Geldermann E., Sharma B.P., Andrade-Piedra J.L., Forbes G.A. (2012). Use of Phosphonate to Manage Foliar Potato Late Blight in Developing Countries. Plant Dis..

[53] Liljeroth E., Bengtsson T., Wiik L., Andreasson E. (2010). Induced resistance in potato to *Phytophthora infestans*-effects of BABA in greenhouse and field tests with different potato varieties. Eur. J. Plant Pathol..

[54] Burra D.D., Berkowitz O., Hedley P.E., Morris J., Resjö S., Levander F., Liljeroth E., Andreasson E., Alexandersson E. (2014). Phosphite-induced changes of the transcriptome and secretome in *Solanum tuberosum* leading to resistance against *Phytophthora infestans*. BMC Plant Biol..

[55] Lim S., Borza T., Peters R.D., Coffin R.H., Al-Mughrabi K.I., Pinto D.M., Wang-Pruski G. (2013). Proteomics analysis suggests broad functional changes in potato leaves triggered by phosphites and a complex indirect mode of action against *Phytophthora infestans*. J. Proteom..

[56] Lobato M.C., Machinandiarena M.F., Tambascio C., Dosio G.A.A., Caldiz D.O., Daleo G.R., Andreu A.B., Olivieri F.P. (2011). Effect of foliar applications of phosphite on post-harvest potato tubers. Eur. J. Plant Pathol..

[57] Flor H.H. (1971). Current Status of the Gene-For-Gene Concept. Annu. Rev. Phytopathol..

[58] Dou D., Kale S.D., Wang X., Chen Y., Wang Q., Wang X., Jiang R.H.Y., Arredondo F.D., Anderson R.G., Thakur P.B. (2008). Conserved C-terminal motifs required for avirulence and suppression of cell death by *Phytophthora sojae* effector Avr1b. Plant Cell.

[59] Whisson S.C., Boevink P.C., Moleleki L., Avrova A.O., Morales J.G., Gilroy E.M., Armstrong M.R., Grouffaud S., Van West P., Chapman S. (2007). A translocation signal for delivery of oomycete effector proteins into host plant cells. Nature.

[60] Haas B.J., Kamoun S., Zody M.C., Jiang R.H.Y., Handsaker R.E., Cano L.M., Grabherr M., Kodira C.D., Raffaele S., Torto-Alalibo T. (2009). Genome sequence and analysis of the Irish potato famine pathogen *Phytophthora infestans*. Nature.

[61] Martin G.B., Bogdanove A.J., Sessa G. (2003). Understandind the Function of Plant Desease Resistance Proteins. Annu. Rev. Plant Biol..

[62] Rodewald J., Trognitz B. (2013). Solanum resistance genes against *Phytophthora infestans* and their corresponding avirulence genes. Mol. Plant Pathol..

[63] Malcolmson J.F., Black W. (1966). New R genes in *Solanum demissum* lindl. And their complementary races of *Phytophthora infestans* (Mont.) de bary. Euphytica.

[64] Black W., Mastenbroek C., Mills W.R., Peterson L.C. (1953). A proposal for an international nomenclature of races of *Phytophthora infestans* and of genes controlling immunity in *Solanum demissum* derivatives. Euphytica.

[65] Helgeson J.P., Pohlman J.D., Austin S., Haberlach G.T., Wielgus S.M., Ronis D., Zambolim L., Tooley P., McGrath J.M., James R.V. (1998). Somatic hybrids between *Solanum bulbocastanum* and potato: A new source of resistance to late blight. Theor. Appl. Genet..

[66] Van Der Vossen E.A.G., Gros J., Sikkema A., Muskens M., Wouters D., Wolters P., Pereira A., Allefs S. (2005). The Rpi-blb2 gene from *Solanum bulbocastanum* is an Mi-1 gene homolog conferring broad-spectrum late blight resistance in potato. Plant J..

[67] Park T.H., Gros J., Sikkema A., Vleeshouwers V.G.A.A., Muskens M., Allefs S., Jacobsen E., Visser R.G.F., Van Der Vossen E.A.G. (2005). The late blight resistance locus Rpi-blb3 from *Solanum bulbocastanum* belongs to a major late blight R gene cluster on chromosome 4 of potato. Mol. Plant-Microbe Interact..

[68] Park T.-H., Vleeshouwers V.G.A.A., Hutten R.C.B., Van Eck H.J., Van Der Vossen E., Jacobsen E., Visser R.G.F. (2005). High-resolution mapping and analysis of the resistance locus Rpi-abpt against *Phytophthora infestans* in potato. Mol. Breed..

[69] Ballvora A., Ercolano M.R., Weiß J., Meksem K., Bormann C.A., Oberhagemann P., Salamini F., Gebhardt C. (2002). The R1 gene for potato resistance to late blight (*Phytophthora infestans*) belongs to the leucine zipper/NBS/LRR class of plant resistance genes. Plant J..

[70] Van Der Vossen E., Sikkema A., Te Lintel Hekkert B., Gros J., Stevens P., Muskens M., Wouters D., Pereira A., Stiekema W., Allefs S. (2003). An ancient R gene from the wild potato species *Solanum bulbocastanum* confers broad-spectrum resistance to *Phytophthora infestans* in cultivated potato and tomato. Plant J..

[71] Huang S., Van Der Vossen E.A.G., Kuang H., Vleeshouwers V.G.A.A., Zhang N., Borm T.J.A., Van Eck H.J., Baker B., Jacobsen E., Visser R.G.F. (2005). Comparative genomics enabled the isolation of the R3a late blight resistance gene in potato. Plant J..

[72] Foster S.J., Park T.H., Pel M., Brigneti G., Sliwka J., Jagger L., Van Der Vossen E., Jones J.D.G. (2009). Rpi-vnt1.1, a Tm-22 homolog from *Solanum venturii*, confers resistance to potato late blight. Mol. Plant-Microbe Interact..

[73] Pel M.A., Foster S.J., Park T.H., Rietman H., Ven Arkel G., Jones J.D.G., Van Eck H.J., Jacobsen E., Visser R.G.F., Van Der Vossen E.A.G. (2009). Mapping and cloning of late bright resistance genes from *Solanum venturii* using an interspecific candidate gene approach. Mol. Plant-Microbe Interact..

[74] Vossen J.H., van Arkel G., Bergervoet M., Jo K.R., Jacobsen E., Visser R.G.F. (2016). The *Solanum demissum* R8 late blight resistance gene is an Sw-5 homologue that has been deployed worldwide in late blight resistant varieties. Theor. Appl. Genet..

[75] Jiang R., Li J., Tian Z., Du J., Armstrong M., Baker K., Tze-Yin Lim J., Vossen J.H., He H., Portal L. (2018). Potato late blight field resistance from QTL dPI09c is conferred by the NB-LRR gene R8. J. Exp. Bot..

[76] Raffaele S., Farrer R.A., Cano L.M., Studholme D.J., MacLean D., Thines M., Jiang R.H.Y., Zody M.C., Kunjeti S.G., Donofrio N.M. (2010). Genome evolution following host jumps in the irish potato famine pathogen lineage. Science.

[77] Vleeshouwers V.G.A.A., Oliver R.P. (2014). Effectors as tools in disease resistance breeding against biotrophic, hemibiotrophic, and necrotrophic plant pathogens. Mol. Plant-Microbe Interact..

[78] Du Y., Weide R., Zhao Z., Msimuko P., Govers F., Bouwmeester K. (2018). RXLR effector diversity in *Phytophthora infestans* isolates determines recognition by potato resistance proteins; the case study AVR1 and R1. Stud. Mycol..

[79] Yin J., Gu B., Huang G., Tian Y., Quan J., Lindqvist-Kreuze H., Shan W. (2017). Conserved RXLR effector genes of *Phytophthora infestans* expressed at the early stage of potato infection are suppressive to host defense. Front. Plant Sci..

[80] Aguilera-Galvez C., Champouret N., Rietman H., Lin X., Wouters D., Chu Z., Jones J.D.G., Vossen J.H., Visser R.G.F., Wolters P.J. (2018). Two different R gene loci co-evolved with Avr2 of *Phytophthora infestans* and confer distinct resistance specificities in potato. Stud. Mycol..

[81] Park T.H., Vleeshouwers V.G.A.A., Huigen D.J., Van Der Vossen E.A.G., Van Eck H.J., Visser R.G.F. (2005). Characterization and high-resolution mapping of a late blight resistance locus similar to R2 in potato. Theor. Appl. Genet..

[82] Restrepo S., Myers K.L., Del Pozo O., Martin G.B., Hart A.L., Buell C.R., Fry W.E., Smart C.D. (2005). Gene profiling of a compatible interaction between *Phytophthora infestans* and *Solanum tuberosum* suggests a role for carbonic anhydrase. Mol. Plant-Microbe Interact..

[83] Bradshaw J.E., Bryan G.J., Lees A.K., McLean K., Solomon-Blackburn R.M. (2006). Mapping the R10 and R11 genes for resistance to late blight (*Phytophthora infestans*) present in the potato (*Solanum tuberosum*) R-gene differentials of Black. Theor. Appl. Genet..

[84] Solomon-Blackburn R.M., Stewart H.E., Bradshaw J.E. (2007). Distinguishing major-gene from field resistance to late blight (*Phytophthora infestans*) of potato (*Solanum tuberosum*) and selecting for high levels of field resistance. Theor. Appl. Genet..

[85] Brugmans B., Wouters D., Van Os H., Hutten R., Van Der Linden G., Visser R.G.F., Van Eck H.J., Van Der Vossen E.A.G. (2008). Genetic mapping and transcription analyses of resistance gene loci in potato using NBS profiling. Theor. Appl. Genet..

[86] Tan M.Y.A., Hutten R.C.B., Celis C., Park T.H., Niks R.E., Visser R.G.F., Van Eck H.J. (2008). The RPi-mcd1 locus from *Solanum microdontum* involved in resistance to *Phytophthora infestans*, causing a delay in infection, maps on potato chromosome 4 in a cluster of NBS-LRR genes. Mol. Plant-Microbe Interact..

[87] Rauscher G., Simko I., Mayton H., Bonierbale M., Smart C.D., Grünwald N.J., Greenland A., Fry W.E. (2010). Quantitative resistance to late blight from *Solanum berthaultii* cosegregates with RPi-ber: Insights in stability through isolates and environment. Theor. Appl. Genet..

[88] Stewart H.E., Bradshaw J.E., Pande B. (2003). The effect of the presence of R-genes for resistance to late blight (*Phytophthora infestans*) of potato (*Solanum tuberosum*) on the underlying level of field resistance. Plant Pathol..

[89] Poland J.A., Balint-Kurti P.J., Wisser R.J., Pratt R.C., Nelson R.J. (2009). Shades of gray: The world of quantitative disease resistance. Trends Plant Sci..

[90] Ruocco M., Ambrosino P., Lanzuise S., Woo S.L., Lorito M., Scala F. (2011). Four potato (*Solanum tuberosum*) ABCG transporters and their expression in response to abiotic factors and *Phytophthora infestans* infection. J. Plant Physiol..

[91] Ghislain M., Byarugaba A.A., Magembe E., Njoroge A., Rivera C., Román M.L., Tovar J.C., Gamboa S., Forbes G.A., Kreuze J.F. (2019). Stacking three late blight resistance genes from wild species directly into African highland potato varieties confers complete field resistance to local blight races. Plant Biotechnol. J..

[92] Oliva R.F., Cano L.M., Raffaele S., Win J., Bozkurt T.O., Belhaj K., Oh S.K., Thines M., Kamoun S. (2015). A recent expansion of the RXLR effector gene Avrblb2 is maintained in global populations of *Phytophthora infestans* indicating different contributions to virulence. Mol. Plant-Microbe Interact..

[93] Hao D., Yang J., Long W., Yi J., VanderZaag P., Li C. (2018). Multiple R genes and phenolic compounds synthesis involved in the durable resistance to *Phytophthora infestans* in potato cv. Cooperation 88. Agri Gene.

[94] Abiola O., Angel J.M., Avner P., Bachmanov A.A., Belknap J.K., Bennett B., Blankenhorn E.P., Blizard D.A., Bolivar V., Brockmann G.A. (2003). The nature and identification of quantitative trait loci: A community’s view. Nat. Rev. Genet..

[95] Leonards-Schippers C., Gieffers W., Schafer-Pregl R., Ritter E., Knapp S.J., Salamini F., Gebhardt C. (1994). Quantitative resistance to *Phytophthora infestans* in potato: A case study for QTL mapping in an allogamous plant species. Genetics.

[96] Berdugo-Cely J., Valbuena R.I., Sánchez-Betancourt E., Barrero L.S., Yockteng R. (2017). Genetic diversity and association mapping in the Colombian Central Collection of *Solanum tuberosum* L. Andigenum group using SNPs markers. PLoS ONE.

[97] Santa J.D., Berdugo-Cely J., Cely-Pardo L., Soto-Suárez M., Mosquera T., Galeano C.H.M. (2018). QTL analysis reveals quantitative resistant loci for *Phytophthora infestans* and *Tecia solanivora* in tetraploid potato (*Solanum tuberosum* L.). PLoS ONE.

[98] Luo Z.W., Hackett C.A., Bradshaw J.E., McNicol J.W., Milbourne D. (2000). Predicting parental genotypes and gene segregation for tetrasomic inheritance. Theor. Appl. Genet..

[99] Sundaresha S., Sharma S., Shandil R.K., Sharma S., Thakur V., Bhardwaj V., Kaushik S.K., Singh B.P., Chakrabarti S.K. (2018). An insight into the downstream analysis of RB gene in F1 RB potato lines imparting field resistance to late blight. Funct. Plant Biol..

[100] Xu J., Wang J., Pang W., Bian C., Duan S., Liu J., Huang S., Jin L., Qu D. (2013). The potato *R10* resistance specificity to late blight is conferred by both a single dominant *R* gene and quantitative trait loci. Plant Breed..

[101] Vert G., Walcher C.L., Chory J., Nemhauser J.L. (2008). Integration of auxin and brassinosteroid pathways by Auxin Response Factor 2. Proc. Natl. Acad. Sci. USA.

[102] Ravikumar B., Sarkar S., Davies J.E., Futter M., Garcia-Arencibia M., Green-Thompson Z.W., Jimenez-Sanchez M., Korolchuk V.I., Lichtenberg M., Luo S. (2010). Regulation of mammalian autophagy in physiology and pathophysiology. Physiol. Rev..

[103] Koch A., Biedenkopf D., Furch A., Weber L., Rossbach O., Abdellatef E., Linicus L., Johannsmeier J., Jelonek L., Goesmann A. (2016). An RNAi-Based Control of *Fusarium graminearum* Infections through Spraying of Long dsRNAs Involves a Plant Passage and Is Controlled by the Fungal Silencing Machinery. PLoS Pathog..

[104] Quattrocchio F., Verweij W., Kroon A., Spelt C., Mol J., Koes R. (2006). PH4 of petunia is an R2R3 MYB protein that activates vacuolar acidification through interactions with basic-helix-loop-helix transcription factors of the anthocyanin pathway. Plant Cell.

[105] Wang M., Thomas N., Jin H. (2017). Cross-kingdom RNA trafficking and environmental RNAi for powerful innovative pre- and post-harvest plant protection. Curr. Opin. Plant Biol..

[106] Qiao Y., Shi J., Zhai Y., Hou Y., Ma W. (2015). Phytophthora effector targets a novel component of small RNA pathway in plants to promote infection. Proc. Natl. Acad. Sci. USA.

[107] Weiberg A., Wang M., Lin F.M., Zhao H., Zhang Z., Kaloshian I., Da Huang H., Jin H. (2013). Fungal small RNAs suppress plant immunity by hijacking host RNA interference pathways. Science.

[108] Cai Q., Qiao L., Wang M., He B., Lin F.M., Palmquist J., Huang S.-D., Jin H. (2018). Plants send small RNAs in extracellular vesicles to fungal pathogen to silence virulence genes. Science.

[109] Qi T., Guo J., Peng H., Liu P., Kang Z., Guo J. (2019). Host-induced gene silencing: A powerful strategy to control diseases of wheat and barley. Int. J. Mol. Sci..

[110] Dubrovina A.S., Kiselev K.V. (2019). Exogenous RNAs for gene regulation and plant resistance. Int. J. Mol. Sci..

[111] Nowara D., Schweizer P., Gay A., Lacomme C., Shaw J., Ridout C., Douchkov D., Hensel G., Kumlehn J. (2010). HIGS: Host-induced gene silencing in the obligate biotrophic fungal pathogen *Blumeria graminis*. Plant Cell.

[112] Jahan S.N., Åsman A.K.M., Corcoran P., Fogelqvist J., Vetukuri R.R., Dixelius C. (2015). Plant-mediated gene silencing restricts growth of the potato late blight pathogen *Phytophthora infestans*. J. Exp. Bot..

[113] Ghag S.B., Shekhawat U.K.S., Ganapathi T.R. (2014). Host-induced post-transcriptional hairpin RNA-mediated gene silencing of vital fungal genes confers efficient resistance against *Fusarium wilt* in banana. Plant Biotechnol. J..

[114] Rosa C., Kuo Y.-W., Wuriyanghan H., Falk B.W. (2018). RNA Interference Mechanisms and Applications in Plant Pathology. Annu. Rev. Phytopathol..

[115] Tricoli D.M., Carney K.J., Russell P.F., McMaster J.R., Graff D.W., Hadden K.C., Himmel P.T., Hubbard J.P., Boeshore M.L., Quemada H.D. (1995). Field evaluation of transgenic squash containing single or multiple virus coat protein gene constructs for resistance to cucumber mosaic virus, watermelon mosaic virus 2, and zucchini yellow mosaic virus. Bio/Technology.

[116] Kamthan A., Chaudhuri A., Kamthan M., Datta A. (2015). Small RNAs in plants: Recent development and application for crop improvement. Front. Plant Sci..

[117] Pixley K.V., Falck-Zepeda J.B., Giller K.E., Glenna L.L., Gould F., Mallory-Smith C.A., Stelly D.M., Stewart C.N. (2019). Genome Editing, Gene Drives, and Synthetic Biology: Will They Contribute to Disease-Resistant Crops, and Who Will Benefit?. Annu. Rev. Phytopathol..

[118] Law Library of Congress (U.S.), Global Legal Research Directorate (2014). Restrictions on Genetically Modified Organisms.

[119] Dubrovina A.S., Aleynova O.A., Suprun A.R., Ogneva Z.V., Kiselev K.V. (2020). Transgene suppression in plants by foliar application of in vitro-synthesized small interfering RNAs. Appl. Microbiol. Biotechnol..

[120] Song X.S., Gu K.X., Duan X.X., Xiao X.M., Hou Y.P., Duan Y.B., Wang J.X., Yu N., Zhou M.G. (2018). Secondary amplification of siRNA machinery limits the application of spray-induced gene silencing. Mol. Plant Pathol..

[121] Åsman A.K.M., Vetukuri R.R., Jahan S.N., Fogelqvist J., Corcoran P., Avrova A.O., Whisson S.C., Dixelius C. (2014). Fragmentation of tRNA in *Phytophthora infestans* asexual life cycle stages and during host plant infection. BMC Microbiol..

[122] EuroBlight. https://agro.au.dk/forskning/internationale-platforme/euroblight/control-strategies/best-practice/.

[123] Fernández-Pavía S.P., Grünwald N.J., Díaz-Valasis M., Cadena-Hinojosa M., Fry W.E. (2004). Soilborne oospores of *Phytophthora infestans* in central Mexico survive winter fallow and infect potato plants in the field. Plant Dis..

